# Bilateral lateral rectus muscle recession versus unilateral lateral rectus recession with medial rectus resection: a 12-month outcome analysis for intermittent exotropia

**DOI:** 10.3389/fopht.2025.1687829

**Published:** 2025-11-18

**Authors:** Li Qi, Xiaoyan Bian, Wei Wang, Jianxin Jia

**Affiliations:** 1 Department of Human Anatomy, School of Basic Medicine and Forensic Medicine, Baotou Medical College, Baotou, China; 2 Department of Pediatric Eye Disease, Boatou Chaoju Eye Hospital, Baotou, China; 3 National Key Clinical Specialty, Weifang Eye Hospital, Weifang Institute of Ophthalmology, Zhengda Guangming Ophthalmology Group, Weifang, China

**Keywords:** intermittent exotropia, bilateral lateral rectus recession, unilateral lateral rectus recession, medial rectus muscle resection, stereopsis

## Abstract

**Background:**

Intermittent exotropia (IXT) is a common form of strabismus, often treated surgically to improve ocular alignment and binocular vision. This study compares the outcomes of two surgical techniques: bilateral lateral rectus recession (BLRR) and unilateral lateral rectus recession combined with medial rectus muscle resection (ULRRMMR), focusing on sensory eye alignment, stereopsis function, and binocular vision recovery.

**Methods:**

This retrospective study included 306 children with intermittent exotropia, assigned to either the BLRR or ULRRMMR group. Participants were evaluated preoperatively and at 1-, 3-, and 12-months post-surgery for sensory alignment, stereopsis, binocular vision, and complications. Statistical analyses were conducted to compare the outcomes between the two groups.

**Results:**

After surgery at one day, overcorrection was observed more frequently in the BLRR group (P = 0.02). A comparison of the two surgical approaches revealed that the BLRR group demonstrated significantly greater improvements in sensory eye alignment (P = 0.009). In contrast, the ULRRMMR group showed superior outcomes in terms of foveal stereopsis (P = 0.01), binocular vision recovery (P = 0.007), and achieving normal eye alignment (P < 0.001) at 12 months. Notably, there was no significant difference in the rate of complications or binocular vision recovery between the two groups at the 12-month follow-up (P = 0.822).

**Conclusion:**

Both BLRR and ULRRMMR are effective treatments for intermittent exotropia, but the BLRR may be a more optimal choice for enhancing sensory alignment, while ULRRMMR technique offers superior recovery in terms of stereopsis, and binocular vision recovery.

## Introduction

Intermittent exotropia (IXT) is a common form of strabismus that often leads to significant functional impairment, including reduced binocular vision and stereopsis. Surgical intervention remains the primary treatment for IXT, with various surgical approaches aimed at restoring ocular alignment and improving sensory function (1–3). Two commonly employed techniques are bilateral lateral rectus recession (BLRR) and unilateral recession combined with medial rectus muscle Resection (ULRRMMR) (4). While both procedures have demonstrated effectiveness in correcting misalignment, their comparative efficacy in terms of sensory eye alignment, stereopsis, and overall functional recovery remains a topic of ongoing debate.

Previous studies have investigated the outcomes of these surgical techniques, with mixed results regarding the degree of improvement in stereopsis and binocular vision (5–8). For instance, BLRR has been traditionally favored for its ability to achieve a stable and long-lasting correction, while ULRRMMR is thought to provide a more controlled adjustment for cases with specific alignment patterns. However, there is limited evidence directly comparing the postoperative recovery profiles of these two approaches, especially with regard to long-term outcomes in sensory eye alignment and stereopsis.

This study aims to provide a comprehensive comparison of these two surgical methods by assessing their impact on sensory alignment, stereopsis function, binocular vision recovery, and complication rates over a 12-month period.

## Methods

### Study design and participants

This retrospective study reviewed 306 children diagnosed with intermittent exotropia (IXT) who underwent surgery between January 2022 and June 2024. The patients were classified into two surgical groups based on the type of surgical technique used: the BLRR group and the ULRRMMR group. All participants received comprehensive ophthalmological evaluations, which included assessments of best-corrected visual acuity (BCVA), slit lamp examination, synoptophore testing, and Random-dot stereogram analysis. The inclusion criteria for the study were as follows (1): diagnosis of intermittent exotropia (IXT) confirmed by two ophthalmologists (2), age of 12 years or younger (3), BCVA of 1.0 or better, and (4) access to high-quality structural and functional brain imaging data. The exclusion criteria included: (1) presence of other types of strabismus, such as constant exotropia with vertical misalignment, (2) history of ocular diseases other than IXT, (3) prior ocular or brain surgeries, (4) severe physical or mental illnesses or brain conditions.

This retrospective study conformed to the tenets of the Declaration of Helsinki and was approved by the Ethics Committee and Institutional Review Board of Baotou Chaoju Eye Hospital.

### Surgical procedure

The BLRR group underwent bilateral recession of the lateral rectus muscles, with the amount of recession determined based on preoperative alignment measurements. In contrast, the ULRRMMR group received a unilateral recession of the lateral rectus muscle combined with Resection of the medial rectus muscle, tailored to the individual’s alignment characteristics.

### Outcome measures

Sensory alignment and binocular function were evaluated using standardized clinical tests at baseline (preoperatively), postoperative day 1, 1 month, 3 months, and 12 months. The spherical equivalent and refractive correction were recorded when applicable. Sensory alignment was assessed with the synoptophore (simultaneous perception and sensory fusion) and classified as: normal, mild deficit, moderate deficit, and severe deficit according to pre-specified synoptophore response criteria (see **Supplementary Table S1** for thresholds).

Stereopsis was measured using standardized stereotests: the Titmus Fly test (near stereopsis), the Random-dot stereogram (Randot) and the Worth 4-Dot test (for binocular fusion/suppression). For stereopsis quantification we prioritized Randot (seconds of arc) as the primary stereopsis metric; Titmus results were recorded in seconds of arc and reported separately. The Worth 4-Dot test was used to assess fusion/suppression status rather than stereoacuity. Foveal (macular) stereopsis was defined as Randot ≤ 60 arcsec, peripheral stereopsis as Randot > 60 arcsec but detectable, and stereopsis deficit as no measurable stereoacuity on Randot.

Binocular vision (simultaneous perception and motor fusion amplitude) was assessed on the synoptophore and by clinical fusional amplitude measurements where available; normal binocular vision was predefined as the ability to demonstrate simultaneous perception and fusion on synoptophore with appropriate fusional amplitudes per age-standardized norms (see **Supplementary Material**).

Ocular alignment was assessed by cover-uncover test and prism and alternate cover test (PACT) at near (33 cm) and distance (6 m) and recorded in prism diopters (PD). Surgical success was defined as achieving normal alignment (residual deviation ≤ 10 PD at distance) at the 12-month follow-up. Normal alignment was defined as residual deviation ≤10 PD at distance.

All outcome measures were recorded by masked examiners where possible. Detailed thresholds and scoring rules for categorical groupings are provided in **Supplementary Table S1**.

### Statistical analysis

The data were analyzed using SPSS Statistics (version 25.0, IBM Corp., Armonk, NY). Continuous variables were expressed as means with standard deviations (SD) or medians with interquartile ranges (IQR), depending on the distribution. Normality of the data was assessed using the Shapiro-Wilk test. For normally distributed data, independent samples t-tests were used to compare continuous variables between the two surgical groups. For non-normally distributed data, the Mann-Whitney U test was applied. Categorical variables were presented as frequencies and percentages, and group comparisons were performed using the chi-square test or Fisher’s exact test when expected frequencies were less than five. Postoperative outcomes at different time points (one, day, 1 month, 3 months, and 12 months) were analyzed using repeated measures analysis of variance (ANOVA) or the non-parametric Friedman test if the data did not meet assumptions for parametric testing. Pairwise comparisons were conducted with the Bonferroni correction to account for multiple testing. All statistical tests were two-tailed, and all analyses were performed with a significant level of 0.05.

## Results

The basic characteristics of the participants in the two surgical groups are summarized in **Table 1**. No significant differences were observed in age or gender between the BLRR group (9.57 ± 2.97 years) and the ULRRMMR group (9.00 ± 2.87 years) (P = 0.122). The proportion of female patients was 48.70% in the BLRR group and 55.10% in the ULRRMMR group (P = 0.258). However, preoperative near and far prism diopter values were significantly higher in the ULRRMMR group (46.47 ± 14.98 and 44.23 ± 16.06, respectively) compared to the BLRR group (39.57 ± 12.09 and 36.07 ± 12.28, respectively) (P < 0.001 for both). Regarding preoperative binocular vision, 38.00% of patients in the BLRR group and 48.39% in the ULRRMMR group had binocular vision (P = 0.067). For sensory eye alignment, the majority of patients in both groups had severe misalignment, with 78.70% in the BLRR group and 80.77% in the ULRRMMR group showing severe sensory misalignment (P = 0.796). Preoperative stereopsis was significantly better in the ULRRMMR group, with 33.97% of patients having severe impairment compared to 52.00% in the BLRR group (P < 0.001).

**Table 1:** Characteristics of all participants.

Indicator	Surgical approach		P-value
	BLRR (n=150)	ULRRMMR (n=156)	
Age (years)	9.57 ± 2.97	9.0 ± 2.87	0.122^a^
Gender (female, %)	73 (48.70%)	86 (55.10%)	0.258
Preoperative near prism diopter	39.57 ± 12.09	46.47 ± 14.98	<0.001
Preoperative distance prism diopter	36.07 ± 12.28	44.23 ± 16.06	<0.001
Preoperative binocular vision (yes)	57 (38.00%)	75 (48.39%)	0.067
Preoperative sensory eye alignment			0.796
Normal	3 (2.00%)	7 (4.49%)	
Mild deficit	8 (5.30%)	9 (5.77%)	
Moderate deficit	21 (14.00%)	14 (8.97%)	
Severe deficit	118 (78.70%)	126 (80.77%)	
Preoperative stereopsis			<0.001
Normal	3 (2.00%)	7 (4.49%)	
Mild deficit	20 (13.33%)	51 (32.69%)	
Moderate deficit	49 (32.67%)	45 (28.85%)	
Severe deficit	78 (52.00%)	53 (33.97%)	

BLRR, bilateral lateral rectus recession; ULRRMMR, unilateral lateral rectus recession combined with medial rectus resection.

The postoperative outcomes on day 1 for the BLRR group and the ULRRMMR group are shown in **Table 2**. No significant difference in sensory eye alignment was found between the two groups (P = 0.456), with similar distributions of mild to moderate alignment defects. However, a higher proportion of patients in the ULRRMMR group achieved foveal (41.03%) stereopsis compared to the bilateral group (33.33%) (P = 0.001). Additionally, the ULRRMMR group demonstrated significantly better binocular vision recovery, with 83.33% of patients achieving binocular vision recovery compared to 67.11% in the BLRR group (P = 0.001). Moreover, the ULRRMMR group also had a higher rate of normal binocular vision recovery (92.31%) versus the BLRR group (83.22%) (P = 0.02), with minimal cases of overcorrection (7.05% in the unilateral group vs. 16.78% in the bilateral group).

**Table 2:** The results of sensory eye alignment, stereopsis, binocular vision, and ocular alignment after surgery at one day.

	Sensory eye alignment		Stereopsis		Binocular vision		Ocular alignment	
BLRR	Normal	13 (8.67%)	Macular fovea	1 (0.67%)	Yes	100 (67.11%)	Normal	124 (83.22%)
	Mild deficit	27 (18.00%)	Macular stereopsis	50 (33.33%)				
	Moderate deficit	49 (32.67%)	Peripheral stereopsis	56 (37.33%)	No	49 (32.89%)	Overcorrection	25 (16.78%)
	Severe deficit	61 (40.67%)	Stereopsis deficit	43 (28.67%)			Undercorrection	0 (0.00%)
ULRRMMR	Normal	27 (17.31%)	Macular fovea	9 (5.77%)	Yes	130 (83.33%)	Normal	144 (92.31%)
	Mild deficit	14 (8.97%)	Macular stereopsis	64 (41.03%)				
	Moderate deficit	57 (36.54%)	Peripheral stereopsis	60 (38.46%)	No	26 (16.67%)	Overcorrection	11 (7.05%)
	Severe deficit	58 (52.00%)	Stereopsis deficit	23 (14.74%)			Undercorrection	1 (0.64%)
P-value	0.456		0.001		0.001		0.02	

BLRR, bilateral lateral rectus recession; ULRRMMR, unilateral lateral rectus recession combined with medial rectus resection.

The postoperative outcomes for both BLRR group and the ULRRMMR group showed significant improvement over long time (**Table 3**). In the BLRR group, the percentage of patients with sensory eye alignment increased from 17.33% at 1 month to 23.33% at 12 months, with a highly significant trend (P < 0.001). The group also exhibited substantial progress in stereopsis function, with the foveal stereopsis improving from 2.00% at 1 month to 20.17% at 12 months (P < 0.001). Binocular vision recovery was notably high, with 84.7% of patients achieving binocular vision at 1 month, increasing to 95.00% at 12 months (P < 0.001). At 12 months, the motor success rate (defined as residual deviation ≤10 PD at distance) was 87.50% in the BLRR group. However, the normal eye alignment rate showed no significant changes during 1 to 12 months after surgery (P = 0.223). Complication rates were minimal in this group, with 97.52% of patients showing no complications at 1 month, suggesting favorable safety and recovery outcomes. Similarly, the ULRRMMR group demonstrated a higher overall rate of sensory eye alignment (92.16% at 1 month to 93.75% at 12 months), and a significant improvement in stereopsis function. At 12 months, 22.22% of patients showed foveal stereopsis, and 75.69% had improved stereopsis compared to 38.06% at 1 month (P < 0.001). The recovery of binocular vision was even more favorable in this group, with 96.08% of patients achieving binocular vision at 12 months. The motor success rate in the ULRRMMR group was 91.18% at 12 months, which was comparable to the BLRR group (P = 0.281). There was no significant difference in the normal eye alignment from 1 to 12 months (P = 0.779). The complication rates were low in the unilateral recession group, with minimal undercorrection (6.25% at 12 months) and only a small increase in overcorrection (3.92% at 1 month to 5.88% at 12 months).

**Table 3:** Changes of sensory eye alignment and stereopsis after 1-, 3, and 12-month.

	Sensory eye alignment					Stereopsis				
		One month	Three months	12 months	P value		One month	Three months	12 months	P value
BLRR	Normal	26 (17.33%)	18 (12.16%)	28 (23.33%)	<0.001	Macular fovea	3 (2.00%)	11 (7.38%)	24 (20.17%)	<0.001
	Mild deficit	38 (25.33%)	40 (27.03%)	40 (33.33%)		Macular stereopsis	89 (59.33%)	106 (71.14%)	73 (61.34%)	
	Moderate deficit	52 (34.67%)	69 (46.62%)	37 (30.83%)		Peripheral stereopsis	0 (0.00%)	23 (15.44%)	15 (12.61%)	
	Severe deficit	34 (22.67%)	21 (14.19%)	15 (12.50%)		Stereopsis deficit	58 (38.67%)	9 (6.04%)	7 (5.88%)	
ULRRMMR	Normal	1 (0.65%)	7 (4.58%)	14 (9.66%)	<0.001	Macular fovea	0 (0.00%)	4 (2.61%)	32 (22.22%)	<0.001
	Mild deficit	22 (14.29%)	41 (26.80%)	43 (29.66%)		Macular stereopsis	59 (38.06%)	108 (70.59%)	109 (75.69%)	
	Moderate deficit	111 (72.08%)	89 (58.17%)	74 (51.03%)		Peripheral stereopsis	87 (56.13%)	35 (22.88%)	3 (2.08%)	
	Severe deficit	20 (12.99%)	16 (10.46%)	14 (9.66%)		Stereopsis deficit	9 (5.81%)	6 (3.92%)	0 (0.00%)	
P-value		0.003	0.289	0.009			0.703	0.137	0.01	–

BLRR, bilateral lateral rectus recession; ULRRMMR, unilateral lateral rectus recession combined with medial rectus resection.

When comparing the two surgical approaches (**Table 3**), the BLRR group had significantly higher improvement in sensory eye alignment (P = 0.009). However, ULRRMMR group were more favorable in terms of foveal stereopsis (P = 0.01) and binocular vision recovery (P = 0.007), motor success rate at 12 months (91.18% vs. 87.50%, P = 0.281), and normal eye alignment (P < 0.001) at 12 months. Of note, there was no significant difference in complications binocular vision recovery between the two groups at 12 months (P = 0.822, **Table 4**).

**Table 4:** Changes of binocular vision, ocular alignment, and complication after 1-, 3, and 12-month.

	Binocular vision					Ocular alignment					Complication		
		One month	Three months	12 months	P trend		One month	Three months	12 months	P trend	No	Yes	P value
BLRR	Yes	127 (84.7%)	130 (87.84%)	114 (95.00%)	<0.001	Normal	127 (84.67%)	129 (87.16%)	79 (79.80)	0.223	118 (97.52%)	3 (2.48%)	0.822
	No	23 (15.33%)	18 (12.16%)	6 (5.00%)		Overcorrection	18 (12.00)	13 (8.78%)	12 (12.12%)				
						Undercorrection	5 (3.33%)	6 (4.05%)	8 (8.08%)				
	Total	150 (100.00%)	148 (100.00%)	120 (100.00%)		Total	150 (100.00%)	148 (100.00%)	99 (100.00%)				
ULRRMMR	Yes	148(96.10%)	147 (96.08%)	144 (100.00%)	0.007	Normal	141 (92.16%)	140 (92.11%)	135 (93.75%)	0.779	142 (97.93%)	3 (2.07%)	
	No	6 (3.90%)	6 (3.92%)	0 (0.00%)		Overcorrection	6 (3.92%)	5 (3.29%)	0 (0.00%)				
						Undercorrection	6(3.92%)	7 (4.61%)	9 (6.25%)				
	Total	154 (100.00%)	153 (100.00%)	144 (100.00%)		Total	153 (100.00%)	152 (100.00%)	144 (100.00%)				
P value	–	<0.001	0.008	0.007			0.033	0.133	<0.001				

BLRR, bilateral lateral rectus recession; ULRRMMR, unilateral lateral rectus recession combined with medial rectus resection.

## Discussion

Our study compared the surgical outcomes of BLRR and ULRRMMR for treating strabismus in pediatric patients. The findings reveal that both surgical approaches significantly improved sensory eye alignment, stereopsis, and binocular vision recovery over 12 months. Notably, the ULRRMMR group demonstrated superior outcomes in stereopsis recovery, binocular vision recovery, and normal eye alignment at both short- and long-term follow-ups. However, the BLRR group showed a greater improvement in sensory eye alignment. The low complication rates in both groups affirm the safety and efficacy of these surgical techniques.

In comparison with existing literature, our findings align with and expand upon previous studies on strabismus surgical outcomes. Studies have consistently shown that surgical correction significantly improves binocular vision and stereopsis in children with strabismus (9–11). The choice between bilateral lateral rectus recessions surgery and unilateral recession of a lateral rectus (LR) muscle combined with resection of its antagonist medial rectus (MR) muscle (R&R) for treating basic IXT remains a subject of debate due to differing preferences among surgeons. Bilateral recession is often favored for its ability to preserve horizontal comitance, minimize narrowing of the palpebral fissure, and provide a more aesthetically pleasing result. In contrast, R&R is preferred by some for its single-eye approach, which is more acceptable to patients when only one eye is visibly misaligned, and for its predictability in secondary surgeries, as R&R on the contralateral eye is often more reliable than bilateral medial rectus resections. A study by the Pediatric Eye Disease Investigator Group (PEDIG) (12), compared these two methods and reported suboptimal surgical outcomes within three years in 46% of patients undergoing bilateral recessions and 37% of those undergoing R&R, a 9% difference. However, this difference was not statistically significant, and the study concluded that neither approach could be considered superior. Nevertheless, our observation of superior binocular vision recovery and stereopsis in the ULRRMMR group aligns with these findings, suggesting that the more precise correction achieved through unilateral approaches better restores fusion and depth perception. On the other hand, the improvements in sensory eye alignment observed in the BLRR group are consistent with reports that bilateral procedures are more effective in cases of large or symmetric deviations, as they provide more comprehensive realignment of the visual axes. Furthermore, our study adds to the literature by providing robust evidence on the long-term outcomes of these procedures, showing that the benefits persist and even improve over 12 months. Notably, few studies have directly compared the two techniques, making our results particularly valuable in guiding clinical decisions. Additionally, our study highlights the relatively lower complication rates associated with both techniques, addressing concerns raised in earlier research about the potential risks of overcorrection or undercorrection, which have historically been areas of clinical debate. By offering comparative data, this study bridges a gap in the literature, supporting the tailored selection of surgical approaches based on individual patient needs and preoperative characteristics.

The differential outcomes between the two surgical groups may be attributed to their distinct mechanisms of action (13–15). ULRRMMR, by combining a lateral rectus recession with medial rectus resection in a single eye, not only corrects the misalignment mechanically but also allows for a more targeted modulation of muscle force vectors. Biomechanical studies have suggested that resection increases the active tension of the medial rectus while recession decreases lateral rectus pull, effectively enhancing the balance of agonist–antagonist forces on the operated eye (16, 17). This precise modulation may facilitate restoration of central fusion by aligning the visual axes within Panum’s fusional area, thereby promoting high-grade stereopsis (18). Neurophysiological research indicates that when ocular alignment is restored within the critical spatial limits for foveal correspondence, binocular cortical neurons can be effectively re-engaged, leading to recovery of fine stereopsis and binocular summation (19–21). In contrast, BLRR primarily alters muscle length symmetrically across both eyes, improving gross alignment and comitance but potentially providing less selective restoration of the agonist–antagonist balance at the level of a single eye. As a result, BLRR may yield robust motor alignment but slightly less optimal central sensory fusion, which could explain the observed differences in stereopsis and binocular vision outcomes between the groups.

The study has several strengths, including a large sample size, long-term follow-up, and comprehensive evaluation of sensory and functional outcomes. However, this study has several limitations that should be considered. Firstly, the retrospective design and lack of randomization may introduce selection bias, affecting the comparability of the two surgical groups. The follow-up duration was relatively short, and longer-term follow-up is needed to assess the durability of the outcomes. Potential confounders, such as the severity and duration of exotropia prior to surgery, were not fully controlled, which may impact the results. Additionally, variability in surgical techniques across surgeons and the single-center nature of the study limits the generalizability of the findings. The reliance on subjective outcome measures, like sensory alignment and stereopsis, could also introduce measurement bias, while the absence of quality-of-life assessments restricts a holistic view of the impact on patients (22). Finally, although the sample size was adequate for detecting major outcomes, it may have lacked power to identify smaller differences or rare complications.

## Conclusion

In conclusion, while both surgical techniques offer effective solutions for treating IXT, the ULRRMMR may be superior in terms of achieving binocular vision recovery, stereopsis recovery, and normal eye alignment. However, the BLRR group also showed significant improvements, especially in optimal sensory alignment, suggesting that both approaches are valuable depending on the individual patient’s needs. These results provide valuable insights for clinicians in tailoring surgical interventions to optimize functional and sensory outcomes in pediatric patients.

## Data Availability

The original contributions presented in the study are included in the article/**Supplementary Material**. Further inquiries can be directed to the corresponding authors.

## References

[1] McKean-Cowdin R Cotter SA Tarczy-Hornoch K Wen G Kim J Borchert M . Prevalence of amblyopia or strabismus in Asian and non-hispanic white preschool children: multi-ethnic pediatric eye disease study. Ophthalmology. (2013) 120:2117–24. doi: 10.1016/j.ophtha.2013.03.001, PMID: 23697956 PMC4848013

[2] Pan CW Zhu H Yu JJ Ding H Bai J Chen J . Epidemiology of intermittent exotropia in preschool children in China. Optom Vis Sci. (2016) 93:57–62. doi: 10.1097/OPX.0000000000000754, PMID: 26583796

[3] Govindan M Mohney BG Diehl NN Burke JP . Incidence and types of childhood exotropia: a population-based study. Ophthalmology. (2005) 112:104–8. doi: 10.1016/j.ophtha.2004.07.033, PMID: 15629828

[4] Kassem RR Radwan RE El-Mofty RMA Elhilali HM . Unilateral versus bilateral lateral rectus recession for correction of small to moderate angle exotropia. Int Ophthalmol. (2024) 44:408. doi: 10.1007/s10792-024-03324-1, PMID: 39419908

[5] Mohan K Sharma SK . Long-term ocular alignment and sensory outcomes after medial rectus recession for high AC/A ratio esotropia. J Pediatr Ophthalmol Strabismus. (2024) 61:344–50. doi: 10.3928/01913913-20240508-03, PMID: 38815097

[6] Han M Shen T Wang X Yu X Zhu B Wen Y . Surgical outcomes of bilateral lateral rectus recession versus unilateral recession and resection for the divergence excess type of intermittent exotropia. Indian J Ophthalmol. (2023) 71:3558–62. doi: 10.4103/IJO.IJO_2977_22, PMID: 37870024 PMC10752302

[7] Lee MH Smith DR Kraft SP Wan MJ . Comparison of unilateral versus bilateral lateral rectus recession for small angle intermittent exotropia: outcomes and surgical dose-responses. J Pediatr Ophthalmol Strabismus. (2022) 59:350–5. doi: 10.3928/01913913-20220131-03, PMID: 35192384

[8] Roda M Pellegrini M Rosti A Fresina M Schiavi C . Augmented bimedial rectus muscles recession in acute acquired concomitant esotropia associated with myopia. Can J Ophthalmol. (2021) 56:166–70. doi: 10.1016/j.jcjo.2020.10.006, PMID: 33160920

[9] Zhao G Fu J Qi Y Wang Y Wei W . Six-month binocular stereopsis recovery and its influencing factors in children with intermittent exotropia. BMC Ophthalmol. (2024) 24:139. doi: 10.1186/s12886-024-03412-x, PMID: 38539156 PMC10967032

[10] Pang Y Gnanaraj L Gayleard J Han G Hatt SR . Interventions for intermittent exotropia. Cochrane Database Syst Rev. (2021) 9:CD003737. doi: 10.1002/14651858.CD003737.pub4, PMID: 34516656 PMC8437222

[11] Jiménez-Romo CA Rangel-Padilla A Páez-Garza JH . Timely surgery in intermittent exotropia. J Binocul Vis Ocul Motil. (2023) 73:21–7. doi: 10.1080/2576117X.2022.2152265, PMID: 36622863

[12] Pediatric Eye Disease Investigator Group Writing Committee Donahue SP Chandler DL Holmes JM Arthur BW . A randomized trial comparing bilateral lateral rectus recession versus unilateral recess and resect for basic-type intermittent exotropia. Ophthalmology. (2019) 126:305–17. doi: 10.1016/j.ophtha.2018.08.034, PMID: 30189281 PMC6348023

[13] Kushner BJ . Selective surgery for intermittent exotropia based on distance/near differences. Arch Ophthalmol. (1998) 116:324–8. doi: 10.1001/archopht.116.3.324, PMID: 9514485

[14] Lee SY Hyun Kim J Thacker NM . Augmented bilateral lateral rectus recessions in basic intermittent exotropia. J AAPOS. (2007) 11:266–8. doi: 10.1016/j.jaapos.2007.02.014, PMID: 17572342

[15] Fletcher MC Silverman SJ . Strabismus. I. A summary of 1,110 consecutive cases. Am J Ophthalmol. (1966) 61:86–94. doi: 10.1016/0002-9394(66)90751-3, PMID: 5904382

[16] Saleem QA Cheema AM Tahir MA Dahri AR Sabir TM Niazi JH . Outcome of unilateral lateral rectus recession and medial rectus resection in primary exotropia. BMC Res Notes. (2013) 6:257. doi: 10.1186/1756-0500-6-257, PMID: 23834953 PMC3708763

[17] Morad Y Kowal L Scott AB . Lateral rectus muscle disinsertion and reattachment to the lateral orbital wall. Br J Ophthalmol. (2005) 89:983–5. doi: 10.1136/bjo.2004.051219, PMID: 16024848 PMC1772789

[18] Gibaldi A Canessa A Sabatini SP . The active side of stereopsis: fixation strategy and adaptation to natural environments. Sci Rep. (2017) 7:44800. doi: 10.1038/srep44800, PMID: 28317909 PMC5357847

[19] Freeman RD . 2015 charles F. Prentice medal award lecture: neural organization of binocular vision. Optom Vis Sci. (2017) 94:931–8. doi: 10.1097/OPX.0000000000001116, PMID: 28858046 PMC5624841

[20] Huang Y Liu Z Wang M Gao L Wu Y Hu J . Cortical reorganization after optical alignment in strabismic patients outside of critical period. Invest Ophthalmol Vis Sci. (2023) 64:5. doi: 10.1167/iovs.64.11.5, PMID: 37535007 PMC10408769

[21] Xi S Zhou Y Yao J Ye X Zhang P Wen W . Cortical deficits are correlated with impaired stereopsis in patients with strabismus. Neurosci Bull. (2023) 39:1039–49. doi: 10.1007/s12264-022-00987-7, PMID: 36481975 PMC10313621

[22] Oh JS Jung JH Shin HJ . Quality of life in intermittent exotropia for Korean children and their parents. BMC Ophthalmol. (2023) 23:185. doi: 10.1186/s12886-023-02919-z, PMID: 37101193 PMC10134591

