# Supplementary material for: Bilateral lateral rectus muscle recession versus unilateral lateral rectus recession with medial rectus resection: a 12-month outcome analysis for intermittent exotropia
Source: Front Ophthalmol (Lausanne). 2025 Nov 18;5:1687829. doi: 10.3389/fopht.2025.1687829 (PMC12669004; doi:10.3389/fopht.2025.1687829)
Supplement: Supplementary file 1 [file Table1.docx]

**Supplementary Table S1. Criteria and thresholds used for classification of binocular visual function.**

| **Test / Domain** | **Measured Function** | **Parameter or Unit** | **Normal** | **Mild Deficit** | **Moderate Deficit** | **Severe Deficit / Absent** |
| --- | --- | --- | --- | --- | --- | --- |
| **Worth 4-Dot Test (W4D)** | Sensory fusion / suppression | Response pattern (4-dot perception at 33 cm and 6 m) | Fusion response (4 dots perceived correctly at both near and distance) | Fusion at near only, suppression at distance | Suppression at both near and distance, alternating responses | Constant suppression or diplopia (no fusion) |
| **Synoptophore (Simultaneous perception & sensory fusion)** | Simultaneous perception, sensory fusion amplitude | Degrees (°) of fusion range | Fusion range ≥ 8° base-out and ≥ 4° base-in | Fusion range reduced by <50% of normal values | Fusion range reduced by ≥50% | No measurable fusion / constant suppression |
| **Randot Stereo Test** | Quantitative stereopsis (fine depth perception) | Seconds of arc (arcsec) | ≤ 60 arcsec (normal fine stereopsis) | 80–200 arcsec (slightly reduced) | 400–800 arcsec (moderately reduced) | > 800 arcsec or unable to perceive disparity (absent stereopsis) |
| **Titmus Fly / Circle Test** | Near stereopsis (gross and contour-based) | Seconds of arc (arcsec) | ≤ 60 arcsec | 80–200 arcsec | 400–800 arcsec | > 800 arcsec or no stereopsis |
| **Cover–uncover and prism alternate cover test (PACT)** | Motor alignment (ocular deviation) | Prism diopters (PD) | ≤ 10 PD (orthotropia / small phoria) | 11–20 PD (mild deviation) | 21–40 PD (moderate deviation) | > 40 PD (severe / constant strabismus) |
| **Composite binocular vision grading*** | Integrated evaluation of sensory alignment, stereopsis, and fusion | Based on combined findings from above tests | All parameters within “normal” range | One domain mildly reduced | ≥2 domains moderately reduced | Severe deficit in ≥1 domain or absence of measurable stereopsis/fusion |
